# Kaposi Sarcoma in an Allograft Kidney Presenting as Acute Kidney Injury With No Cutaneous Lesions: A Case Report

**DOI:** 10.7759/cureus.100689

**Published:** 2026-01-03

**Authors:** Samuel Srinivasan, Sarine Tahmazian, Bipin Ghimire, Rebecca Chacko

**Affiliations:** 1 Internal Medicine, Henry Ford Health, Detroit, USA; 2 Hematology and Oncology, Henry Ford Health, Detroit, USA

**Keywords:** kaposi sarcoma (ks), kaposi sarcoma treatment, post-renal transplant, renal allograft, visceral kaposi sarcoma

## Abstract

Kaposi sarcoma (KS) is an angioproliferative neoplasm linked to human herpesvirus-8 (HHV-8) and occurs more frequently in immunosuppressed patients, including kidney transplant recipients. While KS typically manifests cutaneously, visceral disease represents an uncommon presentation. Visceral KS involving the allograft kidney in patients with a previous renal transplant is an exceedingly rare finding that has seldom been reported. Herein, we report the case of a 66-year-old man with a history of kidney transplant who was found to have biopsy-confirmed, HHV-8-positive visceral KS involving the renal allograft less than one year post transplant, without cutaneous involvement. The patient’s clinical course was marked by severe vasculature obstruction, bowel ischemia, and sepsis as a result of his disease burden, culminating in death despite prompt withdrawal of immunosuppression, highlighting the rarity and severity of allograft-associated KS. The severe manifestations of this patient's disease underscore the need for heightened vigilance when treating our immunosuppressed population to ensure appropriate and prompt care is given.

## Introduction

Kaposi sarcoma (KS) is an angioproliferative soft tissue sarcoma caused by infection with human herpesvirus-8 (HHV-8). Individuals with weakened immune systems, including older adults, those living with HIV/AIDS, and those receiving immunosuppressive therapy, are at a heightened risk of manifesting KS [1]. 

Kidney transplant recipients require immunosuppressive therapy to prevent allograft rejection, but these drugs also increase post-transplantation malignancy risk, with KS being most frequently reported [2,3]. However, KS typically involves the skin or mucosa, while visceral involvement is not common [2]. Case reports documenting renal allograft KS involvement are rare worldwide, particularly in the United States.

Here, we present the case of a patient with post-transplant visceral KS involving the renal allograft. This case illustrates a rare, advanced manifestation of KS in the absence of cutaneous lesions that was identified less than one year after transplant surgery. We highlight the complexity of diagnosing and treating atypical KS in transplant recipients who require immunosuppressive therapy.

## Case presentation

A 66-year-old male patient with a history of colonic arteriovenous malformations, end-stage renal disease secondary to hypertension, and kidney transplantation was hospitalized for acute kidney injury (AKI), initially attributed to hypovolemia and acute tubular necrosis. Transplant surgery had been performed about 7.5 months previously, and the patient’s post-transplant immunosuppressive therapy included mycophenolate mofetil, tacrolimus, and corticosteroids. Kidney biopsy analysis did not show signs of transplant rejection, and abdominal imaging was unremarkable; therefore, he was discharged after one week, after an improving trend in kidney function was observed.

Approximately one week after discharge, at a follow-up visit in the transplant clinic, the patient’s kidney function was markedly worse, with a high serum creatinine level of 7.47 mg/dL (baseline 1.6-1.9 mg/dL), low estimated glomerular filtration rate (eGFR) at 7 mL/minute/m^2^, and critically high blood urea nitrogen at 116 mg/dL (Table 1). The patient was subsequently directed to the Emergency Department for further evaluation.

**Table 1:** Metabolic panel results BUN, blood urea nitrogen; eGFR, estimated glomerular filtration rate

Component	Unit	Reference Range	Patient Value	Interpretation
Anion gap	Arbitrary units	3-13	13	Normal
BUN	mg/dL	10-25	116	Critically high
Calcium	mg/dL	8.2-10.2	9.5	Normal
Carbon dioxide	mmol/L	21-35	18	Low
Chloride	mmol/L	98-111	101	Normal
Creatinine	mg/dL	<1.28	7.47	High
eGFR	mL/minute/1.73 m^2^	>60	7	Low
Glucose	mg/dL	60-140	100	Normal
Potassium	mmol/L	3.5-5.0	5.1	Normal
Sodium	mmol/L	135-145	132	Just below RR

At the time of ED presentation, the patient reported having seen blood in his stool for several days (though his hemoglobin remained stable) and having acute, persistent bilateral lower-extremity edema, more pronounced on the right. He reported adequate hydration and appropriate urine production, and his blood pressure was normal, with systolic blood pressure in the 120-130 mm Hg range. The medical team considered possible causes for the AKI, including hypovolemia due to gastrointestinal bleeding, obstruction secondary to neoplasm/post-transplant lymphoproliferative disorder, medication induced acute tubular necrosis.

To evaluate the reported hematochezia, the patient underwent colonoscopy, which revealed nonspecific, stable findings with no indication for immediate intervention. Imaging was performed to evaluate the edema and AKI. Venography and intravascular ultrasonography revealed severe external compression of the right common iliac vein, for which a venous stent (Abre™ Stent System; Medtronic plc, Galway, Ireland) was placed to restore blood flow.

**Figure 1:**
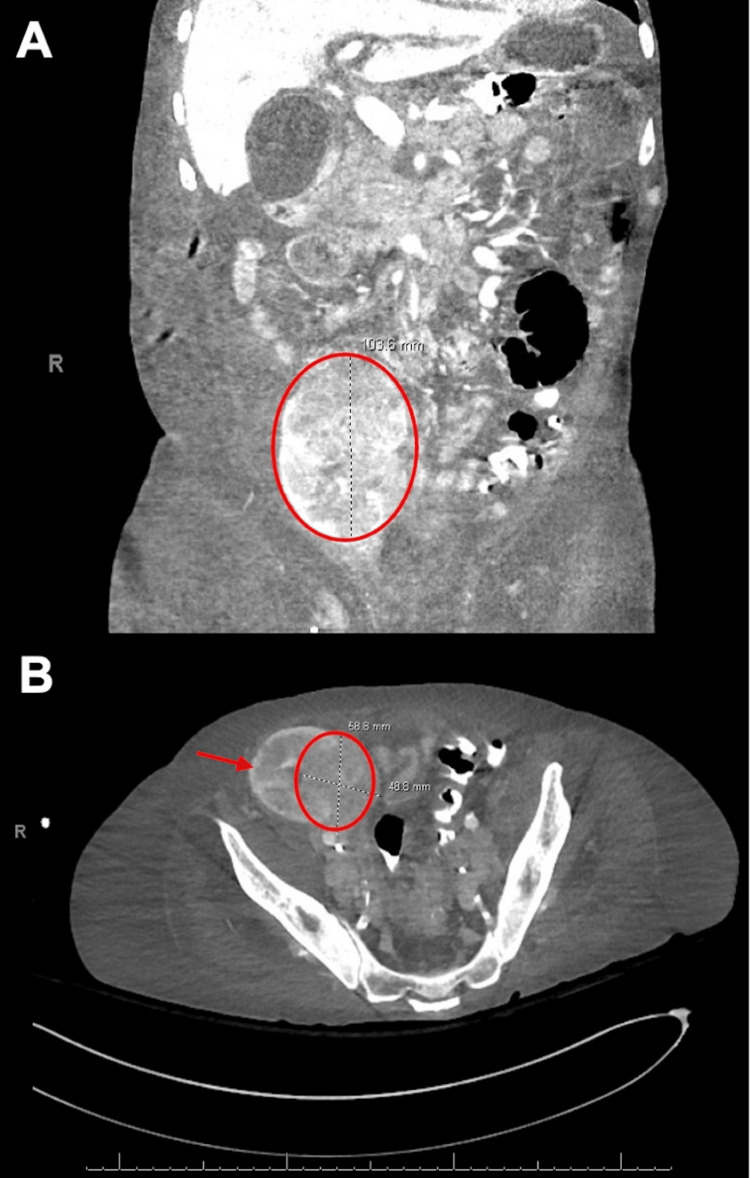
CT abdomen pelvis revealing neoplastic process

Computed tomography (CT) venography of the abdomen and pelvis, optimized to evaluate the renal allograft vasculature, showed a large infiltrative mass centered at the right renal hilum and sinus, extending into the renal allograft parenchyma (Figure 1). The mass showed extrarenal extension invading the right lateral bladder wall, posterior extension encasing the right external iliac vasculature, and superior extension with probable invasion of an adjacent small bowel loop. Extensive retroperitoneal and pelvic lymphadenopathy were also observed. These findings were compatible with the leading differential diagnosis of a neoplastic process causing mechanical obstruction of the urinary outflow tract and surrounding vasculature.

Based on these findings, the patient underwent an interventional radiology-guided biopsy of the transplanted kidney mass. Histopathology revealed KS involving renal parenchyma, and HHV-8 serology results were positive. Importantly, no associated cutaneous lesions were observed. Therefore, the patient’s AKI was believed to be multifactorial, caused by tumor-related external vasculature compression leading to ischemic acute tubular necrosis as well as urinary outflow tract obstruction.

Hemodialysis was resumed, given persistently diminished renal function; however, the patient developed persistent *Enterococcus faecalis* and *Pseudomonas bacteremia* infections and septic shock during hospitalization. Additional complications included small bowel obstruction secondary to a malignant mass effect, which progressed to bowel ischemia. The disease manifestations progressed rapidly, and the patient was transitioned to comfort-focused measures. He died approximately six weeks after admission.

## Discussion

KS classically appears with telltale cutaneous lesions; however, visceral KS involving renal allografts is exceedingly rare. Thus, KS manifesting as AKI or edema with no cutaneous lesions in transplant recipients may be overlooked or misdiagnosed, allowing the disease to progress while patients continue with immunosuppressive therapy. And although KS is one of the most common neoplasms to develop after kidney transplantation, its pathogenesis is not well understood. Additionally, mechanisms driving visceral, rather than cutaneous, involvement are unknown. This report of visceral KS involving the renal allograft is one of the very few documented cases to date that highlight the clinical signs of allograft KS in kidney transplant recipients.

Visceral KS typically occurs within one year of transplantation and is more likely to lead to a poor prognosis than cutaneous KS [2]. Similarly, our patient was diagnosed within one year of transplantation, and his condition rapidly deteriorated. However, our patient’s case was unusual in several regards, including his initial presentation of AKI, which is rarely associated with KS [4]. Additionally, he presented with prominent lower extremity edema, which has been reported in only three other cases of allograft KS [4-6]. But most notably, the extent of our patient’s neoplasm was unusual. The tumor was compressing the bowel blood supply and intestines, which resulted in small bowel obstruction and bowel ischemia, complications that have not been previously reported. Overall, awareness of these atypical manifestations is considered critical for timely recognition of allograft-associated KS in transplant recipients, which can help expedite imaging and further workup. 

Treatment of KS in transplant recipients involves reducing immunosuppression to prevent disease progression [7]. For our patient, despite immediately withholding immunosuppression after the neoplasm was identified, the disease and his decline continued to progress, suggesting an aggressive course of KS. With visceral KS involvement, systemic therapy may be warranted; however, such treatments must be carefully weighed against the patient’s risk of infection, cytopenias, dialysis dependence, and allograft failure. In our patient's case, given his clinical instability, systemic therapy was not appropriate. Multidisciplinary input from oncology, infectious disease, and transplant nephrology teams is essential to optimize patient outcomes.

Approximately 95% of patients who develop KS after transplantation are HHV-8 seropositive [8]. In the current case, given the patient's positive HHV-8 serology results, he likely had latent HHV-8 infection before transplantation, and immunosuppressive therapy may have reactivated the virus, resulting in KS. Some studies have shown that certain immunosuppressants, such as cyclosporine, are associated with higher KS risk [9]. Our patient was treated with tacrolimus, mycophenolate mofetil, and corticosteroids, yet still developed KS. This suggests that any potent immunosuppressive regimen in patients with HHV-8 infection may trigger KS. While screening for HHV-8 infection is not routine, this could be considered to identify patients at risk for developing KS after kidney transplantation.

## Conclusions

Kidney transplantation remains the only curative therapy for patients with end-stage renal disease; however, immunosuppressive therapy can increase the risk of infections, neoplasms, and reactivation of latent viral infections. This case illustrates a rare case of allograft-associated KS and the unique ways in which it can manifest aggressively, such as AKI, lower extremity edema, and bowel obstruction/ischemia in the absence of cutaneous or mucosal lesions in transplant recipients. Increased awareness of these advanced manifestations of KS moving forward may allow expedited diagnostic workup and impact clinical outcomes. 
